# Heart Transplantation from Donors with Takotsubo Cardiomyopathy: Clinical Outcomes and Early Experience from a Single Center

**DOI:** 10.3390/jcm15020842

**Published:** 2026-01-20

**Authors:** Lorenzo Giovannico, Giuseppe Fischetti, Federica Mazzone, Domenico Parigino, Luca Savino, Ilaria Paradiso, Marina Mezzina, Eduardo Urgesi, Claudia Leo, Giuseppe Cristiano, Concetta Losito, Massimiliano Carrozzini, Vincenzo Ezio Santobuono, Andrea Igoren Guaricci, Marco Matteo Ciccone, Massimo Padalino, Tomaso Bottio

**Affiliations:** 1 Cardiac Surgery Unit, Department of Precision and Regenerative Medicine and Ionian Area (DiMePRe-J), Medical School, University of Bari, Piazza Giulio Cesare 11, 70124 Bari, Italy; giuseppe.fischetti@policlino.ba.it (G.F.); federica.mazzone95@gmail.com (F.M.); domenicoparigino@gmail.com (D.P.); lucasavino2905@gmail.com (L.S.); ilariaparadiso2001@gmail.com (I.P.); m.mezzina19@studenti.uniba.it (M.M.); c.leo@studenti.uniba.it (C.L.); giuseppecristiano88@gmail.com (G.C.); dottclosito@libero.it (C.L.); massimiliano.carrozzini@gmail.com (M.C.); massimo.padalino@uniba.it (M.P.); tomaso.bottio@uniba.it (T.B.); 2 University Cardiology Unit, Interdisciplinary Department of Medicine, University of Bari Aldo Moro, 70121 Bari, Italy; eduardourgesi@gmail.com (E.U.); vincenzoezio.santobuono@uniba.it (V.E.S.); andrea.guaricci@gmail.com (A.I.G.); marcomatteo.ciccone@uniba.it (M.M.C.)

**Keywords:** heart transplantation, Takotsubo cardiomyopathy, donor heart, primary graft dysfunction, advanced heart failure, case series

## Abstract

**Background:** Takotsubo cardiomyopathy (TTC) has been historically considered a contraindication for heart donation due to its transient left ventricular dysfunction. However, emerging evidence supports that hearts from donors with fully recovered Takotsubo Cardiomyopathy can be safely transplanted. **Methods:** This case series describes seven heart transplantations performed between January 2022 and September 2025 using donors with previously diagnosed Takotsubo cardiomyopathy. Donor characteristics, intraoperative data, echocardiography data and postoperative outcomes were analyzed. **Results:** The mean donor age was 33.5 years (range 18–58), with a male-to-female ratio of 6:1. All donors exhibited echocardiographic evidence of Takotsubo Cardiomyopathy at the time of brain death, with full or partial recovery before procurement. Coronary angiography excluded obstructive coronary disease. Echocardiographic follow-up demonstrated the mean LVEF increased to 52 ± 6%, reaching 58 ± 4% at 12 months, global longitudinal strain (GLS) improved progressively (from −14.2 ± 2.8% to −18.5 ± 1.9%), confirming normalization of myocardial deformation and the right ventricular function, assessed by TAPSE, rose from 15 ± 3 mm at discharge to 20 ± 2 mm at 12 months. All patients transplanted with donors who had Takotsubo cardiomyopathy are alive at the 12-month follow-up. **Conclusions:** Hearts from donors with resolved Takotsubo Cardiomyopathy can be safely used for transplantation without compromising early- or mid-term outcomes. Expanding donor eligibility criteria to include selected TTC donors may contribute to mitigating organ shortages in advanced heart failure patients.

## 1. Introduction

Heart transplantation remains the gold-standard therapy for patients with end-stage heart failure who are refractory to optimal medical therapy [1]. However, the persistent shortage of suitable donor hearts continues to limit access to heart transplantation and contributes to significant mortality among patients on the waiting list [1,2]. To address this imbalance between demand and supply, the transplant community has progressively explored the use of extended-criteria donors (ECD), including donors with potentially reversible cardiac dysfunction [3,4].

Among the conditions that may cause transient cardiac impairment, Takotsubo cardiomyopathy (TTC)—also known as stress-induced or “broken heart” syndrome—has attracted increasing attention. First described by Sato and colleagues in 1990, TTC is characterized by acute but reversible left ventricular dysfunction in the absence of obstructive coronary artery disease [5]. The condition primarily affects postmenopausal women and is often triggered by emotional or physical stress. Although ventricular function usually recovers completely within days to weeks, acute complications such as heart failure, arrhythmias, and cardiogenic shock can occur [6,7,8].

The pathophysiological mechanisms of TTC remain multifactorial and incompletely understood. The most widely accepted hypothesis involves a catecholamine surge leading to myocardial stunning, microvascular dysfunction, and direct myocyte injury through calcium overload and oxidative stress [9,10]. The distribution of sympathetic nerve endings and β-adrenergic receptor density across the left ventricle explains the different morphological variants—apical, midventricular, basal, and biventricular. More recently, neurocardiac mechanisms and the “brain–heart axis” have been implicated, suggesting that emotional stress, psychiatric disorders, and central nervous system activation play key roles in disease onset and progression [11,12].

Historically, the presence of TTC in potential heart donors has been considered an absolute contraindication due to the concern that myocardial dysfunction, even if reversible, could compromise graft performance after transplantation [13]. Several reports and experimental data suggest that once functional recovery is achieved, myocardial tissue integrity and metabolic capacity are restored to normal levels, making these hearts potentially suitable for transplantation [14,15]. Although recovery of left ventricular ejection fraction represents an eligibility criterion for the vast majority of TTC donors, recent studies indicate that Takotsubo Cardiomyopathy may leave persistent structural, functional, and metabolic abnormalities beyond one year after the acute event, suggesting that echocardiographic normalization does not necessarily equate to full restoration of myocardial physiology. These findings underscore that donor functional recovery should be interpreted within the growing awareness that Takotsubo is a complex and potentially persistent condition, and that acceptance of such hearts should be accompanied by careful evaluation and close follow-up of the recipient [7,16].

The present study reports our single-center experience and represents, to the best of our knowledge, one of the largest single-center cohorts to date evaluating the use of donor hearts affected by Takotsubo cardiomyopathy at the time of brain death, with documented echocardiographic recovery prior to organ procurement. Rather than introducing a novel concept, this study aims to provide confirmatory evidence and systematic follow-up data supporting the feasibility and safety of this approach.

## 2. Materials and Methods

### 2.1. Study Design and Population

This is a retrospective, single-center case series including all heart transplantations performed at the Cardiac Surgery Unit, Policlinico di Bari (Italy), between January 2022 and September 2025, using donor hearts diagnosed with Takotsubo cardiomyopathy (TTC) at the time of brain death.

### 2.2. Donor Selection and Diagnostic Criteria

The diagnosis of Takotsubo cardiomyopathy in potential donors was based on echocardiographic and angiographic findings consistent with the International Takotsubo (InterTAK) diagnostic criteria [1]. Inclusion required:

1. Transient left ventricular wall motion abnormalities extending beyond a single coronary territory (apical, midventricular, or reverse pattern).
2. Absence of significant coronary artery disease on angiography.
3. New ECG changes (ST-segment elevation, T-wave inversion, or QT prolongation) and mild-to-moderate elevation of cardiac biomarkers.
4. Documented recovery of left ventricular function (LVEF ≥ 50%) before organ procurement.

Functional recovery was verified through serial transthoracic echocardiography performed at three time points:

- T0: at hospital admission.
- T1: at brain death confirmation (approximately 24–48 h later).
- T2: immediately prior to organ retrieval (approximately 24 h later).

Only donors showing normalization or near-normalization of wall motion and systolic function at T2 were accepted for transplantation. None of the donors experienced acute complications, such as arrhythmias or cardiogenic shock, during the phase of ventricular dysfunction or throughout the subsequent recovery period. During the study period, approximately 5–6 donors per year with suspected Takotsubo cardiomyopathy were reported; however, some were not utilized due to the lack of timely functional recovery at the T2 time point. According to the National Transplant Center (CNT), Takotsubo cardiomyopathy is not classified among the conditions defining an organ as extended criteria. Informed written consent concerning the use of donors with suspected Takotsubo cardiomyopathy was obtained from all recipients.

### 2.3. Recipient Characteristics and Perioperative Management

Recipients were adults with end-stage ischemic or dilated cardiomyopathy with severe biventricular dysfunction, listed for urgent or elective heart transplantation under national and macro-area priority codes (CNT criteria).

Preoperative assessment included clinical evaluation, laboratory tests, hemodynamic monitoring, and echocardiography. All recipients underwent echocardiographic evaluation at each predefined time point. INTERMACS profiles were assigned at listing.

All patients received standard triple immunosuppression (tacrolimus, mycophenolate mofetil, corticosteroids) and prophylactic antimicrobial therapy according to institutional protocol. Cold static graft preservation using Celsior^®^ (Alpharetta, GA, USA) cardioplegic solution. Patients underwent continuous echocardiographic monitoring and endomyocardial biopsy at 15 days, 21 days, 1 month, and monthly for the first 6 months, and every 2 months up to the first year after heart transplantation for rejection assessment.

### 2.4. Data Collection and Definitions

The following variables were collected for both donors and recipients:

- Demographic data (age, sex, body surface area).
- Comorbidities and risk factors (hypertension, dyslipidemia, diabetes, smoking, peripheral vascular disease).
- Hemodynamic parameters and support requirements (inotropes, ECMO, CVVH, mechanical ventilation).
- Ischemic time, bypass time, and intraoperative events.
- Postoperative outcomes: primary graft dysfunction (PGD), renal replacement therapy, infection, stroke/TIA, in-hospital mortality.

During the preparation of this manuscript, the authors used ChatGPT (GPT-5.2, OpenAI) to assist in the creation of tables, the organization of bibliographic notes, and the standardization of abbreviations. 

### 2.5. Echocardiographic Evaluation

All recipients underwent comprehensive transthoracic echocardiography at 1 week, 1 month, 6 months, and 12 months post-transplant.

Parameters included left ventricular ejection fraction (LVEF, Simpson biplane method), global longitudinal strain (GLS), right ventricular function (TAPSE), and diastolic function indices (E/e′, E/A, and S′ velocities).

Functional recovery was assessed using serial comparisons and expressed as mean ± standard deviation.

## 3. Results

### 3.1. Donor Characteristics

Between January 2022 and September 2025, seven donor hearts affected by Takotsubo cardiomyopathy were utilized for orthotopic heart transplantation.

The mean donor age was 33.5 ± 13.8 years (range 18–58), with a male-to-female ratio of 6:1. Five donors presented the classical apical variant, one the reverse variant, and one the mid-ventricular form of Takotsubo cardiomyopathy. All donors had negative coronary angiography and normal or nearly normal left ventricular ejection fraction (LVEF > 50%) at the time of organ procurement, confirming recovery of ventricular function after initial TTC diagnosis.

All the characteristics of the seven donors are reported in Table 1.

### 3.2. Recipient Characteristics

The seven recipients (five males, two females; mean age 55 ± 9 years, range 38–65) suffered from end-stage ischemic (*n* = 4) or dilated cardiomyopathy (*n* = 3).

According to the INTERMACS classification, three patients were inotrope-dependent (class 3), three had symptoms at rest (class 4), and one was in progressive decline (class 2). Preoperative comorbidities included hypertension (5/7), dyslipidemia (5/7), and diabetes (3/7). Just one recipient required preoperative ECMO and nobody required mechanical ventilation, although three received inotropic support. The mean ICU stay before transplantation was 4 ± 5 days. All the characteristics of the seven donors are reported in Table 2.

### 3.3. Perioperative Findings

All transplants were performed using bicaval orthotopic implantation with standard cold static graft preservation with Celsior^®^ cardioplegia. All procured organs were transported using the Paragonix SherpaPak^®^ System (Waltham, MA, USA). Mean ischemic time was 206 ± 75 min (range 83–280 min). No intraoperative deaths occurred.

All grafts exhibited immediate contractility upon reperfusion. Primary graft dysfunction (PGD) developed in three patients (42.8%), all of whom recovered within 72 h under pharmacological support (dobutamine, epinephrine and norepinephrine) and temporary mechanical assistance (ECMO VA) were required. All cases of primary graft dysfunction were classified as isolated right ventricular PGD according to the ISHLT criteria and required only short-term mechanical circulatory support. The relatively high incidence of PGD observed in this cohort is likely related to our intentional strategy of minimizing catecholamine exposure in recipients of donor hearts with a history of Takotsubo cardiomyopathy, favoring early mechanical support to reduce adrenergic stress and facilitate graft recovery. All the characteristics of the seven donors are reported in Table 3.

### 3.4. Postoperative Outcomes

The mean ICU length of stay was 30 ± 13 days (range 18–61). No patient required renal replacement therapy post-transplant. Two recipients experienced transient infections treated with targeted antibiotics. No episodes of stroke, acute rejection, or chronic graft vasculopathy were recorded during hospitalization. All patients were successfully extubated within 6–48 h (mean 24.6 h, median 24 h), and no in-hospital mortality occurred. At discharge, all recipients had LVEF ≥ 55% and stable hemodynamic profiles. No cases of graft failure, arrhythmia recurrence, or late mortality were observed during the follow-up period. All the characteristics of the seven donors are reported in Table 4.

### 3.5. Echocardiographic Follow-Up

Serial echocardiography and strain analysis were available in all patients.

**Figure 1:**
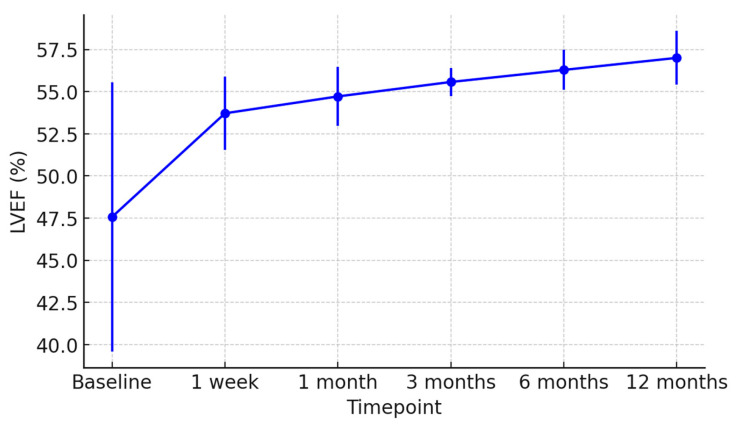
Longitudinal assessment of left ventricular ejection fraction (LVEF) in recipients of hearts from donors with previous Takotsubo cardiomyopathy. Mean LVEF values (±SD) are shown at baseline, 1 week, 1 month, 3 months, 6 months, and 12 months post-transplant, demonstrating progressive improvement and stable graft function over time.

**Figure 2:**
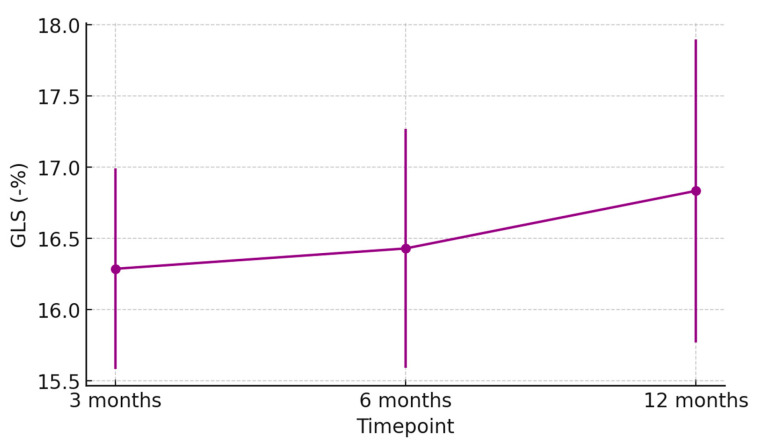
Evolution of global longitudinal strain (GLS) in heart transplant recipients from donors with previous Takotsubo cardiomyopathy. Mean GLS values (±SD) at 3, 6, and 12 months post-transplant show progressive improvement in myocardial deformation, indicating stable and recovering graft function.

**Figure 3:**
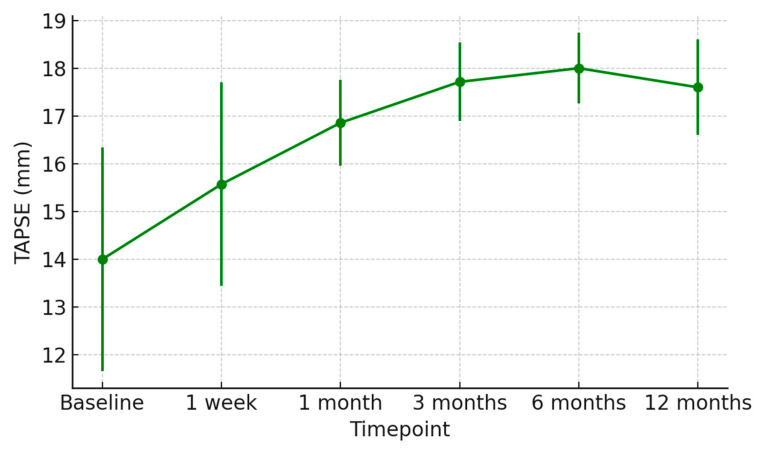
Serial changes in tricuspid annular plane systolic excursion (TAPSE) after heart transplantation from donors with previous Takotsubo cardiomyopathy. Mean TAPSE values (±SD) from baseline to 12 months demonstrate progressive improvement in right ventricular systolic function, indicating favorable graft adaptation over time.

**Figure 4:**
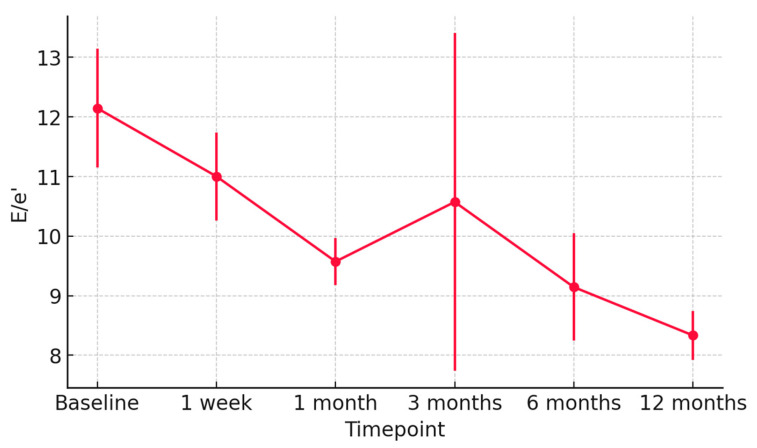
Temporal evolution of the E/e′ ratio in heart transplant recipients from donors with previous Takotsubo cardiomyopathy. Mean E/e′ values (±SD) from baseline to 12 months post-transplant show a progressive decrease, reflecting improved left ventricular diastolic function and normalization of filling pressures over time.

**Figure 5:**
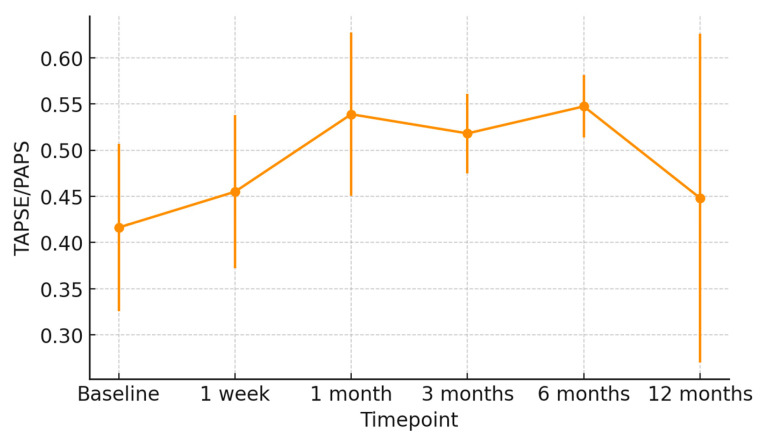
Temporal trend of the TAPSE/PAPS ratio in recipients of hearts from donors with previous Takotsubo cardiomyopathy. Mean TAPSE/PAPS values (±SD) are shown from baseline to 12 months post-transplant, illustrating progressive improvement in right ventricular–pulmonary arterial coupling and overall graft functional recovery.

**Figure 6:**
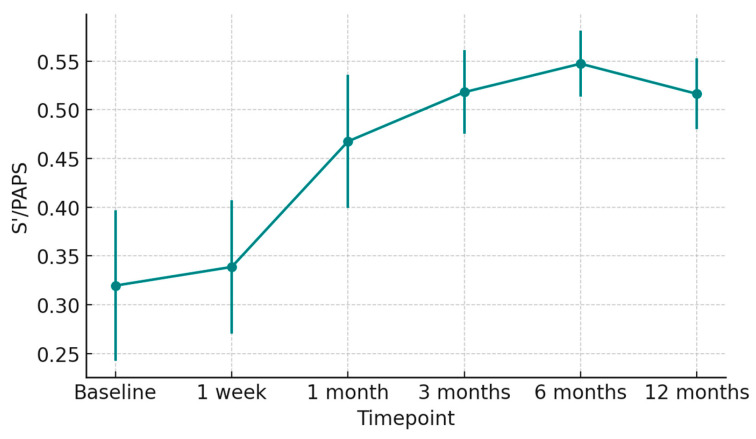
Serial trend of the S′/PAPS ratio after heart transplantation from donors with previous Takotsubo cardiomyopathy. Mean S′/PAPS values (±SD) from baseline to 12 months post-transplant indicate progressive improvement in right ventricular–pulmonary arterial coupling, suggesting enhanced systolic performance and adaptive remodeling of the graft.

At 1 week post-transplant, the mean LVEF increased to 52 ± 6%, reaching 58 ± 4% at 12 months (Figure 1). Global longitudinal strain (GLS) improved progressively (from −14.2 ± 2.8% to −18.5 ± 1.9%), confirming normalization of myocardial deformation (Figure 2). Right ventricular function, assessed by TAPSE, rose from 15 ± 3 mm at discharge to 20 ± 2 mm at 12 months (Figure 3), while E/e′ ratio decreased from 12.8 ± 3.5 to 8.9 ± 2.1, indicating improved diastolic filling pressures (Figure 4). The TAPSE/PAPS ratio increased from 0.32 ± 0.08 at discharge to 0.45 ± 0.06 at 12 months, indicating progressive improvement in right ventricular–pulmonary arterial coupling and graft adaptation (Figure 5). Similarly, the S′/PAPS ratio rose from 0.29 ± 0.05 to 0.41 ± 0.04, confirming restoration of longitudinal right ventricular contractility and efficient ventricular–arterial interaction (Figure 6).

**Figure 7:**
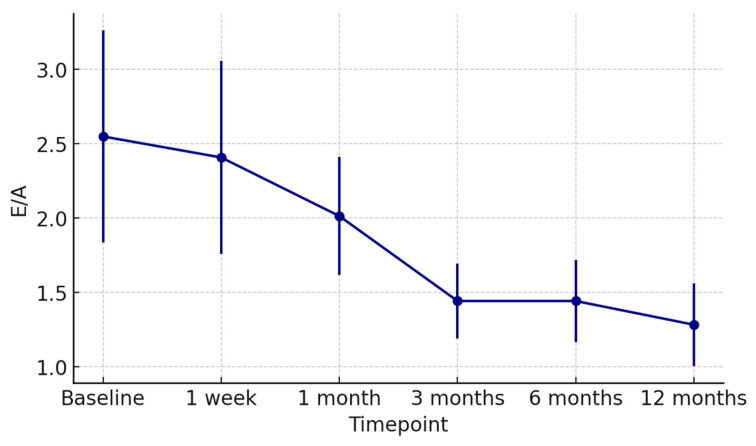
Temporal changes in the E/A ratio after heart transplantation from donors with previous Takotsubo cardiomyopathy. Mean E/A values (±SD) are shown from baseline to 12 months post-transplant, demonstrating a gradual normalization of left ventricular diastolic filling pattern over time.

**Figure 8:**
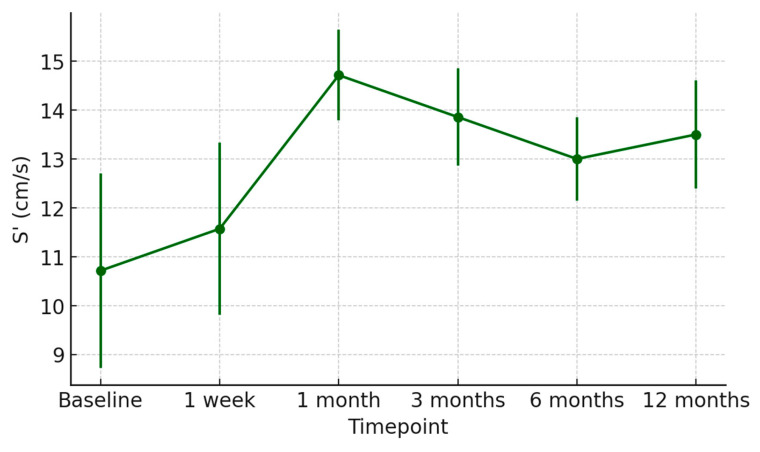
Time-course of systolic tissue Doppler velocity (S′) after heart transplantation from donors with previous Takotsubo cardiomyopathy. Mean S′ values (±SD) from baseline to 12 months post-transplant demonstrate an initial rise and subsequent stabilization, reflecting progressive improvement in longitudinal systolic function of the graft.

Concerning diastolic performance, the E/A ratio improved from 0.78 ± 0.14 at discharge to 1.23 ± 0.18 at 12 months, reflecting normalization of left ventricular filling dynamics (Figure 7). The S′ velocity increased from 7.1 ± 1.2 cm/s to 9.4 ± 1.0 cm/s over the same period, consistent with progressive recovery of longitudinal systolic function (Figure 8).

## 4. Discussion

This study describes one of the largest European single-center experiences with heart transplantation from donors affected by Takotsubo cardiomyopathy (TTC). Our results demonstrate that hearts from donors with documented functional recovery after TTC can be safely transplanted, with excellent early and mid-term outcomes. No mortality or graft failure occurred during hospitalization or follow-up, and all patients achieved normalization of ventricular function at 12 months. Rigorous donor evaluation remains essential. Serial echocardiography, hemodynamic stability, and coronary angiography are critical to exclude underlying ischemic or structural pathology. Functional recovery should be confirmed prior to organ acceptance.

From an ethical perspective, expanding donor criteria to include reversible TTC hearts may significantly reduce waiting list mortality without compromising recipient safety [15]. Donor intracranial hemorrhage is a well-recognized trigger of transient myocardial dysfunction. Early studies reported a higher incidence of early graft dysfunction in this setting, without an adverse impact on long-term survival [17]. More recently, a large propensity score–matched UNOS registry analysis confirmed that hearts from donors with intracranial bleeding are not associated with inferior post-transplant survival when appropriately selected [18]. These data provide important context for our findings, supporting the concept that transient donor ventricular dysfunction reflects a reversible neurocardiogenic process rather than irreversible myocardial injury. El Battrawy et al. [6] and Ghadri et al. [7] reported that TTC-induced injury is characterized by reversible catecholamine toxicity, with minimal necrosis and full recovery of sarcomeric function. More recently, small case reports and series, such as those by Madan et al. (2020) [13], confirmed that donor hearts with recovered TTC function can be used successfully, showing outcomes comparable to standard donors. The pathophysiology of TTC-related dysfunction involves a catecholamine surge, β-adrenergic overstimulation, and microvascular spasm leading to myocardial stunning rather than necrosis [10,11,12]. Histopathologic studies confirm that TTC hearts show minimal irreversible damage, explaining the excellent functional recovery once the catecholaminergic storm subsides [7,13,16].

In our cohort, all recipients showed excellent graft performance, rapid weaning from inotropes, and short ICU stays compared with standard benchmarks [19].

Echocardiographic follow-up revealed progressive improvement in LVEF, GLS, and TAPSE, consistent with stable graft recovery. These findings are in line with other published series, which report comparable 1-year survival and lower incidence of chronic rejection [20,21,22].

Therefore, TTC donor hearts may represent a safe and valuable resource when functional recovery is verified and careful donor–recipient matching is applied.

### Limitations and Future Directions

This study is limited by its retrospective design and small sample size, inherent to the rarity of TTC donors. Furthermore, histologic confirmation of myocardial integrity was not available.

Future multicenter prospective studies and registry-based analyses will be required to validate these findings and to define standardized criteria for TTC donor acceptance.

Long-term follow-up beyond one year is also essential to confirm the absence of late complications such as chronic allograft vasculopathy or graft dysfunction.

## 5. Conclusions

Hearts from donors with reversible Takotsubo cardiomyopathy can be safely used for transplantation, provided that full or near-complete functional recovery is documented prior to procurement. Early and mid-term outcomes are comparable to those obtained from conventional donors.

These findings highlight the importance of re-evaluating donor exclusion criteria and suggest that the use of recovered Takotsubo donor hearts may represent a viable strategy to expand the donor pool and address the ongoing shortage of cardiac grafts.

## Figures and Tables

**Table 1:** Donor characteristics and ischemic times.

Patient	1	2	3	4	5	6	7
BSA (m^2^)	2	2	2	2	2	1	2
Ischemic time	278	280	240	111	240	216	83
Age	32	23	44	18	31	58	27
Sex	f	m	m	m	m	m	m
Ipotropes	on	on	on	on	on	no	no
Dyslipidemia	no	no	no	no	no	no	no
CAD	no	no	no	no	no	no	no
Hypertension	no	no	no	no	no	no	no
Diabetes	no	no	no	no	no	no	no

**Table 2:** Preoperative characteristics of recipients.

Patient (Sex)	1 (m)	2 (m)	3 (m)	4 (f)	5 (m)	6 (f)	7 (m)
Age	51	65	57	38	56	58	57
Diagnosis	Post Isch.	Post Isch.	CMD	CMD	Post Isch.	Post Isch.	CMD
Dyslipidemia	yes	yes	yes	none	yes	none	yes
Hypertension	yes	yes	yes	none	yes	none	yes
Diabetes	none	yes	none	yes	yes	none	none
Peripheral vascular disease	yes	none	none	none	yes	yes	none
naive/redo	naive	naive	naive	naive	redo	naive	naive

**Table 3:** Preoperative support and recipient status.

Patient	1	2	3	4	5	6	7
ECMO pre-op	none	none	none	none	none	ECMO pre-op	none
Renal function	**G3a**	**G4-5**	**G1**	**G2**	**G3b**	**G1**	**G2**
Inotropes pre-op	none	inotropes	inotropes	none	inotropes	none	none
Mechanical ventilation pre-op	none	none	none	none	none	none	none
CVVH PRE-OP	none	none	none	none	none	none	none
Bilirubin (mg/dL)	1	3	2	1	1	2	1
ICU stay (days) pre-op	0	0	0	0	14	11	0
INTERMACS class	4. symptoms at rest	3. inotrope dependent	3. inotrope dependent	4. symptoms at rest	3. inotrope dependent	2. progressive decline	4. symptoms at rest
WL status	2A	1B	1B	2A	1B	1A	2A

**Table 4:** Postoperative outcomes and follow-up results.

Patient	1	2	3	4	5	6	7
ICU length of stay (days)	61	33	32	18	31	19	20
ECMO post-op	ECMO post-op	ECMO post-op	none	none	ECMO post-op	none	none
CVVH post-op	none	none	none	CVVH post-op	none	CVVH post-op	none
Stroke/TIA post-op	none	none	none	none	none	none	none
Infection post-op	Infection post-op	none	none	none	none	none	none
Intubation time (hours)	24	24	6	22	48	24	24
Primary graft disfunction	PGD post-op	PGD post-op	none	none	PGD post-op	None	none
Oral tacrolimus	on	on	on	on	on	on	on
Oral mycophenolate	on	on	on	on	on	on	on
Dialysis	no	no	no	no	no	no	no
eGFR_30	no	no	no	no	no	no	no

## Data Availability

The data presented in this study are available upon request from the corresponding author.

## Abbreviations

The following abbreviations are used in this manuscript:

ACS	Acute Coronary Syndrome
BSA	Body Surface Area
CAD	Coronary Artery Disease
CMD	Dilated Cardiomyopathy
CNT	Centro Nazionale Trapianti (Italian National Transplant Center)
CVVH	Continuous Venovenous Hemofiltration
E/A	Early-to-Late Diastolic Filling Velocity Ratio
E/e′	Early Mitral Inflow Velocity to Early Diastolic Mitral Annular Velocity Ratio
ECMO	Extracorporeal Membrane Oxygenation
ECD	Extended Criteria Donor
EF	Ejection Fraction
GLS	Global Longitudinal Strain
ICU	Intensive Care Unit
INTERMACS	Interagency Registry for Mechanically Assisted Circulatory Support
IQR	Interquartile Range
ISHLT	International Society for Heart and Lung Transplantation
LV	Left Ventricle/Left Ventricular
LVEF	Left Ventricular Ejection Fraction
PAPS	Pulmonary Artery Systolic Pressure
PGD	Primary Graft Dysfunction
SD	Standard Deviation
S′	Systolic Tissue Doppler Velocity
TAPSE	Tricuspid Annular Plane Systolic Excursion
TIA	Transient Ischemic Attack
TTC	Takotsubo Cardiomyopathy (Stress-Induced Cardiomyopathy)
TTE	Transthoracic Echocardiography
WL	Waiting List
